# Questions on the Legal Claims of Apothecaries

**Published:** 1812-01

**Authors:** 


*To the Editors of the Medical and Physical Journal.*

GENTLEMEN,

THE late decision of the cause, *Fuller* versus *the Executors of the Duke of Queensbury, deceased*, and the observation of the judge thereupon, that apothecaries have no *legal* claim to compensation for their trouble in attending patients, other than as they are paid for their medicines; have given rise to the following queries, which fall within the scope of your correspondent Observator's remarks, p. 386.

1st. Have apothecaries residing in the country a *legal* right to make a charge for *visits* or *journies*, separate from their charge for medicines?

2dly. Have apothecaries residing in London a *legal* right to make a charge of *coach-hire*, for the purpose of visiting their patients?

3dly. If so, have they not an equal right to charge coach-hire, when they visit their patients in their own carriages?

Very few apothecaries are aware of the situation in which they *legally* stand with regard to practising medicine; it would therefore be of general utility to this branch of practitioners to understand:

1st. Whether apothecaries in general have any *legal* right to practise medicine; that is to say, to visit patients in diseases, and to prescribe for their complaints.

2dly. Whether the charter of the Society of the Apothecaries authorises the members of that society to visit patients and to prescribe for them.

3dly. Whether members of the College of Surgeons have a *legal* right to practise as apothecaries, or to visit in, and to prescribe for, any other diseases than those which are strictly surgical.

4thly. Whether members of the College of Surgeons, acting as apothecaries, could substantiate a claim in a court of justice for attendance on patients, where the complaint was not strictly surgical.

5thly. Whether the Royal College of Physicians possess the power of suspending from practice, except for mal-practice, any surgeon, or surgeon-apothecary, whether a member of the *Society of Apothecaries* or not.

As

As the subject of medical reform has lately been much discussed in your Journal, I apprehend you will not think the above queries impertinent to that subject, and that either yourselves or some of your well-informed correspondents, will give such information as will let the apothecaries know precisely how they *legally* stand with regard to their charges and attendance.

A correct report of the above trial in your Journal, would, I conceive, be useful as a matter of reference to that branch of the medical profession who only practise as apothecaries. I am, &c.

